# Evaluating Maternal Health Services Within the Reproductive, Maternal, Newborn, Child Health and Adolescents (RMNCH+A) Framework Amidst the COVID-19 Pandemic in Rural India: A Comprehensive Mixed-Methods Analysis

**DOI:** 10.7759/cureus.55680

**Published:** 2024-03-06

**Authors:** Anuj Mundra, Arjunkumar Jakasania, Abhishek Raut, Swati Misra, Pramod V Bahulekar, Subodh S Gupta, Bishan Garg

**Affiliations:** 1 Community Medicine, Mahatma Gandhi Institute of Medical Sciences, Sevagram, IND

**Keywords:** mixed methods, emerging disease, outbreak, resilient health systems, family planning, rmnch

## Abstract

Background

Around half of the pregnant women in India do not receive full antenatal care. During the year 2020, routine health services were further affected by COVID-19. This study was conducted to assess the effect of the pandemic on the delivery/utilization of reproductive, maternal, newborn, child health, and adolescent (RMNCH+A) services.

Methodology

The study, conducted in Wardha district, Maharashtra, from July to December 2020, aimed to assess maternal health. In Wardha block, 200 pregnant and postnatal women were surveyed using a multistage sampling approach. Adequate knowledge was gauged through Mother and Child Protection Card comprehension. Health system data for April to December 2020 was compared with 2019 district-wide. In-depth interviews were conducted with beneficiaries, including pregnant and post-natal women and healthcare workers. The qualitative inquiries involved medical officers, supervisory staff, community health officers, an auxiliary nurse and midwife (ANMs), Taluka Health Officers, and focus group discussions with accredited social health activists (ASHA), Anganwadi workers (AWW), and Village Health Nutrition and Sanitation Committee members.

Results

Essential services were delivered to both antenatal and postpartum women, though family planning services and health education were the worst affected. Among the survey respondents, 75% of the post-partum women were not using any contraceptives. District-wide coverage of post-abortion/MTP contraception fell by around 90% as compared to the previous year. The most common difficulties faced by the respondents in availing of the services were related to finances and arranging transport to visit health facilities.

Conclusion

Learning from the current pandemic for system strengthening, adequate manpower, and planning to prevent disruption of essential services and promoting e-health and m-health initiatives may prevent such catastrophic events in the future from affecting the delivery of routine services.

## Introduction

India witnesses around 25 million pregnancies every year. To cater to the health care needs of this cohort, the Government of India runs the Reproductive, Maternal, Newborn, Child, and Adolescent Health (RMNCH+A) program, focusing on a strategic approach for establishing the continuum of care across life stages and all levels. Pregnant women, children, and adolescents are vulnerable to many adversities, and especially those already at a disadvantage would be the most affected by the pandemic [1].

India has a maternal mortality ratio (MMR) of about 113 per 100,000 live births. One of the reasons for the high MMR is the low utilization of maternal health services, with only 58% of pregnant women having at least 4 ANC (antenatal care) visits, 44% of women consuming at least 100 IFA (ion-folic acid) tablets during pregnancy, and over 10% of deliveries taking place outside health care facilities [2,3]. These indicators are prone to worsening in the event of any encumbrance of the existing weak public health system.

During the year 2020, a novel coronavirus disease (COVID-19) grappled the globe and, within a few weeks, spread across many countries [4]. India reported its first case on January 30, 2020, and like many other countries, the government imposed a nationwide lockdown beginning March 24, with only essential and emergency services being allowed [5,6].

The pandemic and lockdown were expected to disrupt routine healthcare services due to various factors, including resource allocation to COVID-19, infrastructure repurposing, financial barriers, lockdown-induced movement restrictions, and fear among beneficiaries and healthcare workers (HCW) [7]. Among developing nations such as India, low public expenditure on health and existing disparities in healthcare access and quality within the country make such countries more vulnerable to the aftereffects of such pandemics [8,9].

Considering the long-lasting implications of disruptions of RMNCH+A services, international organizations are advocating for strategies to ensure continued routine healthcare service provision, especially for the most vulnerable segments of society [10,11]. The Government of India released a guidance note for the continuation of essential health services, which envisages reorganization of service delivery, telemedicine, developing alternate models for outreach activities, etc. [12]. The present study was thus conducted to assess the status of delivery and utilization of maternal health services in Wardha District during the COVID-19 pandemic and lockdown period, barriers and enabling factors in delivery and utilization of maternal health services, psychosocial effects, and stigma surrounding the pandemic.

## Materials and methods

Study design 

We adopted a cross-sectional design consisting of qualitative as well as quantitative components. The quantitative part comprised secondary data analysis as well as primary data collection, with a focus on the number of services provided or utilized, such as the number of ANCs registered, home visits, institutional deliveries, episodes of maternal morbidity and mortality, immunization, etc. The qualitative part focused on studying the barriers and enabling factors in delivering/utilizing these services.

Study setting

The study was conducted in Wardha district in the Vidarbha region of Maharashtra. There are eight administrative blocks in the district, and the study was conducted in Wardha block, which has four primary health centers (PHCs), one district hospital (DH), and two medical colleges.

Study duration

The study was conducted from July to December 2020.

Study participants

The study participants included beneficiaries of RMNCH+A services, frontline workers such as accredited social health activists (ASHA), auxiliary nurse midwives (ANM), Anganwadi Workers (AWW), lady health visitors (LHV)/supervisors, and other key informants. These informants encompassed taluka health officers (THO), gynecologists, pediatricians from medical colleges and district hospitals, primary health center (PHC) medical officers (MO), as well as staff from district hospitals. Additionally, key village health nutrition and sanitation committee (VHNSC) members and community health officers (CHOs) contributed to the study, offering valuable insights and perspectives.

Sampling

Quantitative Study

As per NFHS 4, the proportion of women availing of at least four ANC visits in Wardha was 77%, and those getting postnatal care were 85%. However, amidst widespread reports of severe interruptions in routine services, these numbers were bound to reduce. We calculated a sample size of 97 with the assumption that these proportions would be 50% with 10% precision. This was then multiplied by two to account for the design effect of cluster sampling, thus giving us a sample size of 194 for each group (making it 200 for simple calculation). Thus, we aimed to conduct a quantitative survey with 200 pregnant women (PW) and postnatal women (PNC) each. We used a multistage sampling approach in which first we identified 40 clusters (villages) through the probability proportional to population size (PPPS) method and then identified five respondents from each cluster randomly from the list obtained through frontline workers. The respondents were administered the semi-structured questionnaire in the local language (Marathi). Ration cards issued by the government under the public distribution system were used to determine socio-economic status as yellow cards for those below the poverty line (BPL), and white cards for those above the poverty line (APL) [13]. Knowledge regarding the use of the mother and child protection card (MCPC) was considered adequate if a woman was able to explain four domains of MCPC, viz., danger signs, dietary advice, and child/mother immunization. We also compared the health system data regarding maternal health services for the whole district for April-December 2020 with the data for April-December 2019 to understand the situation in the whole district.

Qualitative Inquiry

Three in-depth interviews (IDIs) were also conducted with pregnant and postnatal women from each PHC. The beneficiaries were selected purposefully from different villages to represent different strata of society as well as different villages concerning their distance from the PHC.

We also conducted qualitative inquiries with HCW, which included IDIs with PHC-MO-4, supervisory staff of PHC-4, CHO-8, ANMs-8, THO-1, and focused group discussions (FGDs) with ASHA and AWW-4 each, VHNC-8.

Data entry and analysis

All the information was collected and entered directly on Android tablets using the open data kit (ODK) platform for quantitative surveys. Qualitative inquiries were conducted in the local language, voice recording was done, and transcripts were prepared and translated into English. Content analysis (inductive) was done to identify the themes and extract the relevant data for analysis.

Ethics approval and consent to participate: The study was approved by the institutional ethics committee of the Mahatma Gandhi Institute of Medical Sciences via letter No. MGIMS/IEC/COMMED/90/2020. A written informed consent was obtained from all the respondents before the administration of the questionnaire. Audio recording of the qualitative inquiries was done after obtaining verbal consent from the respondents.

## Results

Quantitative surveys

We interviewed 200 PWs and 200 PNCs. The mean age of PW and PNC women was 25 and 26.3 years, respectively. The mean years of completed education were 11.3 and 11.6 years for PWs and PNCs, respectively. Around half of the PW and two-thirds of PNCs belonged to APL families. The PNCs were interviewed after a mean of 14.5 weeks post-delivery (Table 1).

**Table 1:** Sociodemography of the participants APL: above poverty line, BPL: below poverty line #Antenatal Care ##Postnatal Care @Antyodaya Anna Yojana

	ANC^#^ women (n=200)	PNC^##^ women (n=200)
Age in years (Mean±SD)	25.0 (3.8)	26.3 (7.8)
Completed years of education (Mean±SD)	11.3 (2.7)	11.6 (3.1)
Time since delivery in weeks (Median [IQR])		16 (7 - 21)
Type of ration card (number, %)		
Above poverty line (APL)	104 (52.0)	134 (66.3)
Below poverty line (BPL)	81 (40.5)	52 (25.7)
Antyodayee^@^	2 (1.0)	5 (2.5)
Don't have	13 (6.5)	11 (5.4)

Antenatal care

Among the PW, almost an equal number of primigravida and multigravida women participated. Almost half were in their third trimester at the time of the interview. Almost all of the pregnancies were intended and registered. However, around 15% of them were registered after the first trimester, including 1.5% in the third trimester. Even though 95% of the women were provided with MCPC, only 57% had adequate knowledge regarding its content and use. Around 91% of women were visited at least once by any frontline worker, whereas all the pregnant women had at least one antenatal care (ANC) visit at a healthcare facility. Around 95% of them received DT/TT doses, and about 89% received 180 IFA tablets during the pregnancy. About one-fourth of them did not receive supplementary food, i.e., a take-home ration (THR), from the Anganwadi centers (AWCs) (Table 2).

**Table 2:** Antenatal care and services UPT: Urine pregnancy test, THR: Take-home ration, PHC: Primary health center, CHC: Community health center, VHND: Village health & nutrition day, ANC: Antenatal care, IUGR: Intrauterine growth restriction, RTI: Reproductive tract infection, STI: Sexually transmitted infection, USG: Ultrasound sonography @Uterine Pregnancy Kit # Tetanus-Diphtheria Vaccine * total adds to more than 100% as multiple responses were recorded

Variable (n=200)		Number (%)	95% Confidence Interval (CI)
Pregnancy order	Primigravida	98 (49.0)	0.43-0.55
	Multigravida	102 (51.0)	0.45-0.57
Trimester of pregnancy at the time of interview	1^st^	15 (7.5)	0.04-0.11
	2^nd^	85 (42.5)	0.36-0.49
	3^rd^	100 (50.0)	0.43-0.57
Registration of pregnancy done	Yes	196 (98.0)	0.96-0.99
	No	4 (2.0)	0.00-0.04
Gestational age at registration	≤ 12 weeks	167 (83.5)	0.79-0.87
	13 – 20 weeks	26 (13.0)	0.09-0.17
	> 20 weeks	3 (1.5)	0.00-0.03
Place of pregnancy detection	At home using UPT^@^ kits	92 (46.0)	0.39-0.53
	Private practitioner (Allopathy)	51 (25.5)	0.19-0.32
	Medical college	17 (8.5)	0.05-0.12
	PHC/CHC	14 (7.0)	0.03-0.11
	District Hospital	13 (6.5)	0.03-0.09
	Sub Centre/ VHND (Village Health & Nutrition Day)	13 (6.5)	0.03-0.09
Provided with MCP card	Yes	189 (94.5)	0.91-0.98
	No	11 (5.5)	0.02-0.09
Knowledge regarding use of MCP card (n=189)	Adequate	109 (57.7)	0.48-0.67
	Inadequate	80 (42.3)	0.33-0.52
	Primigravida	53 (66.3)	0.55-0.78
	Multigravida	27 (33.7)	0.22-0.45
At least 1 ANC visit	Yes	200 (100.0)	0.97-1.00
Any Antenatal Investigations	Yes	193 (96.5)	0.93-0.99
	No	7 (3.5)	0.00-0.07
Td^#^ injection	Yes	190 (95.0)	0.91-0.99
	No	10 (5.0)	0.01-0.09
At least 1 Home visit by ASHA/ANM	Yes	183 (91.5)	0.87-0.96
	No	17 (8.5)	0.04-0.13
IFA tablets provided	Yes	177 (88.5)	0.86-0.91
	No	23 (11.5)	0.09-0.14
Take home ration	Provided	153 (76.5)	0.72-0.81
	Not provided	47 (23.5)	0.19-0.28
Attended mothers meeting	Yes	23 (11.5)	0.07-0.16
	No	177 (88.5)	0.84-0.93
Reason for not attending mothers meeting (n=177)*	AWC closed due to COVID	54 (30.5)	0.24-0.38
	Mothers meeting not conducted due to COVID	32 (18.1)	0.12-0.24
	Did not have information about mothers meeting	68 (38.4)	0.31-0.46
	Did not go to AWC due to fear of COVID	16 (9.0)	0.05-0.13
	Was not called for mothers meeting	10 (5.6)	0.03-0.09
	Was not at home at the time of meeting	2 (1.1)	0.00-0.03
Counselled regarding danger signs in pregnancy	Yes	85 (42.5)	0.35-0.49
	No	115 (57.5)	0.50-0.65
Counselled regarding nutrition in pregnancy	Yes	163 (81.5)	0.75-0.88
	No	37 (18.5)	0.12-0.25
Counselled regarding birth preparedness (for 3^rd^ trimester ANC; n=100)	Yes	63 (63.0)	0.53-0.73
	No	37 (37.0)	0.27-0.47
Counselled regarding exclusive Breastfeeding (for 3^rd^ trimester ANC; n=100)	Yes	46 (46.0)	0.36-0.56
	No	54 (54.0)	0.44-0.64
Counselled regarding newborn danger signs (for 3^rd^ trimester ANC; n=100)	Yes	35 (35.0)	0.26-0.44
	No	65 (65.0)	0.56-0.74
Counselled regarding Post-partum contraception (for 3^rd^ trimester ANC; n=100)	Yes	51 (51.0)	0.41-0.61
	No	49 (49.0)	0.39-0.59
Any complications/medical issues during pregnancy	Yes	34 (17.0)	0.12-0.22
	No	166 (83.0)	0.78-0.88
Type of complications* (n=34)	RTI/STI	17 (50.0)	0.34-0.66
	Anemia	2 (5.9)	0.01-0.17
	Antepartum hemorrhage	1 (2.9)	0.00-0.10
	Generalized pain	1 (2.9)	0.00-0.10
	Hyperemesis	7 (20.6)	0.09-0.32
	cervical incompetence	1(2.9)	0.00-0.10
	hypothyroidism	1 (2.9)	0.00-0.10
	IUGR	1 (2.9)	0.00-0.10
	Pain abdomen	3 (8.8)	0.02-0.24
	Pedal oedema	2 (5.9)	0.01-0.17
Consult any health care provider for complications (n=34)	Yes	28 (82.4)	0.65-0.99
	No	6 (17.6)	0.01-0.35
Any problems in availing any health services during pregnancy	Yes	47 (23.5)	0.16-0.31
	No	153 (76.5)	0.69-0.84
Type of problems * (n=47)	Problems in arranging/finding transport services	18 (38.3)	0.26-0.51
	Financial issues	17 (36.2)	0.24-0.51
	Delayed hospital visit/No hospital visit due to fear of COVID.	8 (17.0)	0.08-0.29
	Problems in getting USG done	8 (17.0)	0.08-0.29
	availability of services	7 (14.9)	0.07-0.29
	Delayed registration of pregnancy	7 (14.9)	0.07-0.29
	Delayed registration leading to delayed/less THR	6 (12.8)	0.06-0.27
	Delayed/ No benefits of govt. schemes	6 (12.8)	0.06-0.27
	Difficulty in finding related information	2 (4.3)	0.01-0.12

Around 88% of PW did not attend any mother’s meetings, mostly because the meetings were not conducted or due to a lack of information. Health education and counseling were the most affected, as reflected in the suboptimal numbers for almost all matters that are important during pregnancy, viz., nutrition, danger signs, birth preparedness, breastfeeding, contraception, etc. About 17% of women had some medical issues during their pregnancy, and reproductive tract infection (RTI)/sexually transmitted infection (STI) was a major contributor (Table 2). 

Intra- and postnatal care

It was observed that nearly 48% of deliveries were conducted in a tertiary care facility, about 30% in the district hospital, and 18.5% by private practitioners. There were two home deliveries reported among the study participants. The mean birth weight was 2.7 kg, with almost 25% of the newborns having low birth weight (LBW). Early initiation of breastfeeding (EIBF) was practiced in only 56% of the deliveries. However, over 90% of respondents reported that they were counseled regarding exclusive breastfeeding. Although around 55% of the respondents were counseled regarding postpartum contraception, only a fourth of them were using any form of contraception at present (Table 3).

**Table 3:** Post-natal care and services (n=200) PHC: Primary health centers, CHC: Community health center, NVD: Normal vaginal delivery, LSCS: Lower segment caesarean section, PPIUCD: Postpartum intrauterine contraceptive devices, ILI: Influenza-like illness

Variables		Number (%)	95% Confidence Interval (CI)
Place of delivery	Medical college	96 (48)	(42.16,53.84)
	District Hospital	60 (30)	(25.94,34.06)
	Private practitioner	37 (18.5)	(14.65,22.35)
	PHC/CHC	4 (2.0)	(0.47,3.53)
	Sub Centre	1 (0.5)	(0.00,1.23)
	At home	2 (1.0)	(0.00,2.29)
Mode of delivery	NVD	114 (57)	(49.75,64.25)
	LSCS	86 (43)	(35.75,50.25)
Discharge post-delivery [mean days (SD)]	NVD	3.9 (1.7)	-
	LSCS	6.4 (2.9)	-
Birth Weight in Kg (n=200) [mean (SD)]		2.7 (0.5)	-
Low birth weight (n=200)	Yes	50 (25.0)	(19.27,30.73)
	No	150 (75.0)	(69.27,80.73)
Informed regarding New-born danger signs	Yes	89 (44.5)	(38.66,50.34)
	No	111 (55.5)	(49.66,61.34)
Complications after discharge	Yes	4 (2.0)	(0.23,3.77)
	No	196 (98.0)	(96.23,99.77)
IFA received after delivery	Yes	115 (57.5)	(49.76,65.24)
	No	85 (42.5)	(34.76,50.24)
Initiation of breastfeeding	Early (Within 1 hour of birth)	112 (56)	(48.72,63.28)
	Delayed (after 1 hour of birth)	88 (44)	(36.72,51.28)
Delayed initiation of breastfeeding by mode of delivery	NVD (n=114)	33 (28.94)	(21.81,36.07)
	LSCS (n=86)	55 (63.9)	(53.52,74.28)
Counselled regarding Exclusive breastfeeding	Yes	181 (90.5)	(85.56,95.44)
	No	19 (9.5)	(4.56,14.44)
Counselled regarding Post-partum contraception	Yes	111 (55.5)	(49.66,61.34)
	No	89 (44.5)	(38.66,50.34)
Using any post-partum contraceptive	No	146 (73)	(66.06,79.94)
	Yes	54 (27)	(20.06,33.94)
	PPIUCD	11 (20.4)	(11.21,29.59)
	Tubectomy	43 (79.6)	(70.41,88.79)
Received THR from AWC	Yes	167 (83.5)	(77.76,89.24)
	Did not receive at all	3 (1.5)	(0.08,2.92)
	After delivery but not during pregnancy	1 (0.5)	(0.00,1.29)
	During pregnancy but not after delivery	29 (14.5)	(9.76,19.24)
Type of problems (n=48)	Financial issues	15 (31.25)	(19.97,42.53)
	Problems in arranging/finding transport services	16 (33.33)	(21.79,44.87)
	Accessibility issues (services unavailable/available with difficulty)	8 (16.0)	(7.28,24.72)
	Feared visiting hospital due to COVID	4 (8.3)	(1.86,14.74)
	Mother and baby separated due to ILI symptoms/COVID	2 (4.2)	(0.00,9.96)
	Doctors not even touching for examination	2 (4.2)	(0.00,9.96)
	Delayed services	1 (2.1)	(0.00,6.18)

We found that around 83% of the PNCs received THR from AWCs both before and after delivery, about 14.5% received THR during pregnancy but not after delivery, while a small proportion of mothers (0.5%) received it after delivery but not during pregnancy, and 1.5% did not receive THR at all. Only 57.5% of them reported having received IFA tablets after delivery.

Nearly one-fourth of the ANCs and one-fifth of the PNCs reported problems in availing health services. The major problems faced by them were related to arranging transport and finances and delayed or no hospital visits due to fear of COVID-19.

Qualitative inquiries

Based on the qualitative inquiries, we identified six themes.

Services During the COVID-19 Pandemic

It was revealed that most of the beneficiaries received the necessary services during the pandemic and subsequent lockdown, including home visits by frontline workers, immunization, IFA supplements, investigations, etc. However, a few of them also revealed that they did not receive proper counseling and health education, especially for diet, family planning, and self-care during pregnancy, during home visits as well as VHND sessions. According to the majority of health workers, while numerous health programs faced setbacks, antenatal and postnatal services were prioritized among non-COVID services. It was also reported that there was a lack of trust in the health system. The reputation of hospitals also suffered due to rumors that those visiting hospitals would be falsely labeled as COVID-19-positive and admitted. One of the PHC-MO stated, “ANC check-ups were done regularly, but many other government programs had to be stopped during the Corona pandemic period. There was not much effect on the provision of health services to the ANC, PNC, under-five children, and eligible couples. But few had lost trust in hospitals and health workers.”

Benefits of JSY (Janani Suraksha Yojana)

The majority of the eligible respondents reported affirmatively receiving the benefits under JSY.

Differences in Health Services Before and During the Pandemic

The major differences before and during the pandemic were the home visits by the frontline workers, investigations, and long waiting times at health facilities. While both the quality and quantity of home visits were hampered, the respondents were also informed about issues in getting the required investigations done. Also, the waiting times in the health facilities increased during the period.

The supply of take-home ration (THR) from AWCs was also delayed. One of the PNC mothers commented “We experienced a long waiting time for receiving any service. Although there was no change in the behavior of the staff, ANM was busy with COVID-related work, hence they did not pay a home visit. In Anganwadi, food supplements were not provided for the child." One of the ANC women informed us regarding frontline workers that “earlier they were doing all their work with utmost care and were handling everything well, but during the Corona pandemic they had many other things to do; they would provide us information quickly and leave; they were not able to give much time.”

Mental Status

Most of the respondents stated that they experienced stress, anxiety, and loneliness during the lockdown. Some also mentioned feelings of irritability, anger, fear of infection, and disrupted sleep patterns, while others expressed concerns about the future. A few reported being in a generally negative emotional state. One of the respondents stated, "I felt stressed, anxious, and lonely during the lockdown."

Stigma and Fear

A lot of stigma existed in the community regarding COVID-19 initially, mostly against the returning migrants. The infected and quarantined people received minimal support, to the extent that people even stopped talking to them. The health workers reported that, due to fear of contagion, many beneficiaries avoided visiting health facilities and immunization sessions. The fear was evident not only among the beneficiaries but also among the HCWs. One of the PHC-MO stated, “There was an atmosphere of fear due to the pandemic; all the health workers became overcautious. They were worried that they could get infected with Corona. Due to their fear, the beneficiaries initially avoided availing of the services, but later they started visiting us. Women were afraid to come for immunization.”

Difficulties Faced in the Provision of RMNCH+A Services During the COVID-19 Pandemic

One of the PHC-MOs expressed that the availability of ambulances was an issue. Fear and low confidence among HCWs, along with COVID-related activities, hampered routine activities. Some CHOs reported that, apart from occasional medicine stockouts, there were no difficulties in providing services, but the fear of getting infected and transmitting it to family members was in the back of their minds while performing their duties. The beneficiaries had difficulty arranging transportation. One PHC-MO mentioned that “As the ambulance was used for shifting corona cases, most of the time it was not available for RMNCHA services. The health workers were scared about getting infected and wondered how they could continue to work, but later their confidence increased. The staff was engaged in anti-covid activities, which affected routine services. We had problems with the supply of some medicines. The ANC registrations were not delayed, but family planning and counseling were affected. Immunization services were not affected much.”

Secondary data analysis

Table 4 summarizes the comparison of key RMNCH+A indicators achievement against the target between 2019 (April-December) and 2020 (April-December). It suggests a decline in ANC registration by around 5 percent points (i.e., the difference between the two percentages). Overall, the proportion of full antenatal care also declined by about 3% during the pandemic and lockdown period as compared to the previous year. However, achievement in the current year for indicators like tetanus (TT)/ diphtheria (DT) immunization, IFA supplementation, hemoglobin estimation, and PNC check-up visits was similar to the previous year. 

**Table 4:** Comparison of RMNCH+A indicator achievement against target between April and December 2019 and April and December 2020 ANC: Antenatal care, PW: Pregnant women, IFA: Iron-folic acid, PHC: Primary health center, CHC: Community health center, SDH: Subdural hematoma, PPIUCD: Post-partum intrauterine contraceptive device, PAIUCD: post-abortion intrauterine contraceptive device, IUD: intrauterine device # “Full antenatal care” defined as four or more antenatal visits, at least one tetanus toxoid (TT) injection and reported consumption of iron folic acid (IFA) tablets or syrup for a minimum of 100 days [14]. * Against the target for the whole year

	Apr-Dec 2019	Apr-Dec 2020
	Achievement against estimated target % (95% CI)	
ANC care		
ANC Registration*	68.5 (0.59-0.78)	63.9 (0.54-0.73)
ANC Registration within first trimester	88.8 (0.83-0.94)	91.7 (0.87-0.97)
Pregnant women (PW) received 3 ANC	95.8 (0.89-0.98)	97.3 (0.91-1.00)
PW tested for hemoglobin (Hb) 4 or more times in respective ANCs to total ANC registration	98.6 (0.96-1.00)	96.1 (0.93-0.99)
ANC given 180 IFA tablets	97.7 (0.93-0.99)	97.0 (0.91-0.99)
Pregnant women tested for syphilis	36.9 (0.26-0.48)	34.6 (0.23-0.47)
TT2 or booster*	15.6 (0.08-0.27)	16.1 (0.08-0.28)
Full ANC care^#^	65.7 (0.56-0.75)	62.2 (0.52-0.71)
Women receiving 1st post-partum checkup within 48 hours of home delivery	100.0 (1.00-1.00)	100.0 (1.00-1.00)
Number of mothers provided full course of 180 IFA tablets after delivery	90.3 (0.81-0.95)	90.5 (0.82-0.95)
Pregnant women screened for HIV	107.6 (1.0-1.0)	93.0 (0.88-1.00)
Delivery services		
Home deliveries % (out of all deliveries)	0.2 (0.02-0.38)	0.2 (0.02-0.38)
Institutional deliveries % (out of all deliveries)	99.8 (0.62-1.00)	99.8 (0.62-1.00)
Public % (out of all deliveries)	84.2 (0.69-0.99)	76.7 (0.64-0.87)
Private % (out of all deliveries)	15.6 (0.01-0.37)	23.1 (0.02-0.44)
SC deliveries% (out of public institutional deliveries)	0.8 (0.02-0.57)	0.9 (0.03-0.58)
PHC deliveries% (out of public institutional deliveries)	2.9 (0.06-0.88)	3.1 (0.07-0.91)
CHC/SDH deliveries (out of public institutional deliveries)	26.7 (0.17-0.39)	29.0 (0.21-0.43)
DH deliveries (out of public institutional deliveries)	19.4 (0.13-0.28)	25.1 (0.16-0.32)
Medical college deliveries % (out of public institutional deliveries)	50.3 (0.35-0.65)	41.9 (0.31-0.55)
Family planning*		
Male sterilization	7.1 (0.01-0.16)	3.1 (0.00-0.07)
Female sterilization	65.9 (0.58-0.74)	42.3 (0.34-0.51)
Total sterilization	61.0 (0.52-0.69)	39.1 (0.30-0.48)
PPIUCD	58.5 (0.50-0.67)	39.3 (0.29-0.50)
PAIUCD	6.4 (0.02-0.12)	1.0 (0.00-0.04)
Antara	33.7 (0.23-0.44)	6.1 (0.02-0.10)
IUD insertion	30.3 (0.21-0.40)	20.1 (0.13-0.28)
Number of oral pills cycles distributed	457.3 (391.6-523.0)	400.3 (334.3-466.3)

The proportion of deliveries conducted in private institutions increased during the pandemic as compared to the previous year. An increase in deliveries at primary and secondary-level public institutions was seen during the pandemic. While this increase was marginal at sub-centers, PHCs, CHCs, and SDH, the increase in the proportion of deliveries conducted at the district hospital was notable (25% in 2020 as compared to 19% in 2019-20). However, the overall decline in the deliveries conducted in public institutions was mainly due to a 9% decline in the deliveries conducted at medical colleges from the previous year.

Regarding family planning services, it was observed that coverage for both temporary and permanent methods declined during the pandemic as compared to the previous year. Coverage of permanent methods dropped to 39.1% in 2020 as compared to 61% in 2019, whereas coverage of PPIUCD and post-abortion IUCD (PAIUCD) dropped to 39.3% and 1.0% as compared to 58.5% and 6.4%, respectively, in the previous year.

Table 5 summarizes the difference in various RMNCH+A indicators for April-December 2020 compared to the April-December 2019 duration in percentage points for Wardha district. A negative sign signifies that the coverage has declined during April-December 2020, i.e., during the COVID pandemic, as compared to the previous year. 

**Table 5:** Difference in RMNCHA indicators of April 2020 to December 2020 compared to April to December 2019 in percentage points for Wardha district SBA: Skill birth attendant, ECP: Emergency contraceptive pills, MTP: Medical termination of pregnancy, IUCD: Intrauterine contraceptive device, PPIUCD: Postpartum intrauterine contraceptive device, PAIUCD: Post abortion intrauterine contraceptive device, NSV: Non-scalpel vasectomy, ANC: Antenatal care, PNC: Postnatal women $ Difference in other RMNCH+A indicators of Apr-Dec 2020 in percentage compared to April-Dec 2019 indicators (difference in absolute number expressed as percentage change from Apr-Dec 2019)

	Difference in other RMNCH+A indicators ^$^
ANC/PNC services	
Number of Home Deliveries attended by Skill Birth Attendant (SBA) (Doctor/Nurse/ANM)	-11.8
Still Birth	5.8
Abortion (spontaneous)	15.1
MTP up to 12 weeks of pregnancy	-19.5
Number of new-borns weighed at birth	-8.1
Number of New-borns breast fed within 1 hour of birth	-10.7
Women receiving 1st post-partum check-up between 48 hours and 14 days	-8.9
Contraception services	
Number of non-scalpel vasectomy (NSV)/conventional vasectomy conducted	-56.8
Number of laparoscopic sterilizations (excluding post abortion) conducted	-63.4
Number of women provided with post abortion/MTP contraception	-90.4
Number of Interval IUCD Insertions (excluding PPIUCD and PAIUCD)	-33.6
Injectable contraceptive-Antara program- First dose	-86.4
Number of combined oral pill cycles distributed	-12.5
Number of condom pieces distributed	-67.6
Number of emergency contraceptive pills (ECP) given	-67.1

It suggests that many indicators related to maternal health worsened during the pandemic as compared to the previous year. Stillbirths and spontaneous abortions increased by about 5% and 15%, respectively, during the pandemic. Other services like recording birth weight, EIBF, and postpartum check-ups within 2-14 days of home deliveries were also reduced by around 10%. Among family planning services, the hardest hit was post-abortion and injectable contraception, both declining by nearly 90% points; laparoscopic sterilization, condoms, and ECP distribution declined by around two-thirds each; and vasectomy declined to less than half as compared to 2019.

## Discussion

In the present study, we aimed to assess the delivery and utilization of RMNCH+A services during the COVID-19 pandemic and lockdown in rural areas of Wardha district. Although the pandemic did not seem to directly affect maternal outcomes, the indirect effects were many and could be long-lasting. Major services affected were family planning, health education, and counseling, among others, which were affected on a smaller scale. Services through AWCs, such as supplementary food and mother’s meetings, were also affected. The difficulties faced by the beneficiaries in availing themselves of health services were mainly related to financial issues, transportation problems, fear of contagion when visiting hospitals, and long waiting times at health facilities. Stigma, fear, stress, and adverse mental states were also reported by the beneficiaries as well as the providers. Non-COVID health services were affected due to the repurposing of resources, infrastructure, and manpower for COVID-related activities.

During the initial phase of the pandemic in India, there was a decline in patient attendance for non-COVID health conditions due to reasons like initial movement restrictions and fear of getting infected. Moreover, public health activities like VHNDs were canceled on several occasions to avoid such events becoming hotspots of COVID-19 transmission and also due to the utilization of existing resources for containing the spread of COVID. Further, the loss of income due to the nationwide lockdown also prevented people from accessing non-emergency health services to cut on the associated direct and indirect costs [14]. Past experiences have shown the impact of such catastrophic events on sexual and reproductive health is often difficult to measure [11].

In the present study, we found that ANC registration declined by around 5% during the study period as compared to the corresponding period in the previous year. The disruption in RMNCH+A services may lead to an additional 38 million unwanted pregnancies, resulting in increased unsafe abortions, intimate partner violence, RTI/STI, pregnancy-related complications, and maternal and infant morbidity and mortality [15,16]. It is estimated that a meager 10% decline in pregnancy-related services may result in around 28,000 additional maternal deaths and post-natal complications in about 1.7 million women globally. According to another estimate, over 50,000 women in low- or middle-income countries (LMICs) could die due to reduced access to quality health care as an indirect effect of the pandemic [17]. The pandemic may have had a sustained effect on healthcare seeking, as was also seen several months after the Ebola outbreak in African countries during 2013-16 when an additional 3600 maternal and neonatal deaths were observed in Sierra Leone alone [18,19]. Institutional deliveries were not affected much as per our study; however, another study in Uttar Pradesh found them to decline by around 2.2% during March-June 2020, which was the period of comparatively stricter lockdown, i.e., when the services were affected the most [20].

The present study found that family planning services were one of the worst affected during the pandemic. The number of medical termination of pregnancies (MTPs) declined by one-fifth, and spontaneous abortion increased by around 15%. Such deterioration in health indicators was also seen during the Ebola outbreak in several countries in Africa [19,21]. As per estimates, a mere 10% reduction in access to family planning services could result in around 49 million women with unmet needs for contraception and 15 million unintended pregnancies over the next 12 months globally [17].

Health education and promotion is a core strategy to improve health outcomes. It facilitates self-care, provides individuals with the required knowledge, and empowers them to take appropriate health action [22,23]. Universal access to the full antenatal package includes counseling and preparation for newborn care, breastfeeding, birth, and emergency preparedness [22]. Health education and counseling suffered hugely during the pandemic, and most of the women were not provided with vital information on issues related to pregnancy, childbirth, and postpartum care, as per our observations. Fear, as elicited in the current study, both among HCWs and beneficiaries, along with the diversion of HCWs to COVID-related work, could be blamed for the neglect of health education and promotion in the context of non-COVID routine health services [14]. The focus on health education and promotion should never be lost, as it maximally ameliorates the determinants of health among the marginalized and vulnerable groups, that are affected the most during such a catastrophe [24].

In our study, many respondents reported a negative state of mind. Fear of contagion, both horizontal and vertical, limited accessibility of health services, a lack of social support, and isolation or quarantine during the pandemic increased the risk of psychological problems and stress for PWs and PNCs. This can lead to further complications during and after the pregnancy [25-29].

The essence of the study was population-based sampling and triangulation of data from qualitative inquiries and secondary data, which gives high confidence regarding the accuracy of our results. However, we might have missed out on women who were not registered with the fasting laboratory workers (FLWs). Several ANCs were in their third trimester at the time of the interview, and many of them would have gotten registered before the lockdown started, thus showing a better picture than the actual one in our study. The period when the beneficiaries faced the maximum difficulty was April-May 2020. However, we collected the data at a later time, and hence the information given by the beneficiaries may be an underreflection of the actual situation. Our results may not represent remote and hard-to-reach areas where the problems might be manifolds of what we found.

## Conclusions

Improving RMNCH+A services and indicators is a priority for all governments across the globe and international development partners. From the providers’ view, failure to act and prioritize relevant services during the recovery phase of the current crisis would entrench the ill effects and reverse the gains made during the past several decades.

In the wake of the possibility of future waves of COVID and outbreaks of other emerging and re-emerging diseases, the current opportunity must be utilized to reinvigorate health systems and strengthen efforts that focus on decentralized, community-based, and client-focused mechanisms for accessing health services and to build a resilient health system for the future. The development and promotion of e-health/m-health and addressing the needs of vulnerable and marginalized populations to prevent the aftereffects of such adversity from affecting them should be the prime focus of governments across the globe.
